# Plaque index and gingival index during rapid maxillary expansion of patients with unilateral cleft lip and palate

**DOI:** 10.1590/2177-6709.22.6.043-048.oar

**Published:** 2017

**Authors:** Maria Olívia Rocha, Dauro Douglas Oliveira, Fernando Oliveira Costa, Laíze Rosa Pires, Amanda Rafaela Diniz, Rodrigo Villamarim Soares

**Affiliations:** 1 Pontifícia Universidade Católica de Minas Gerais, Programa de Pós-graduação em Odontologia (Belo Horizonte/MG, Brazil).; 2 Universidade Federal de Minas Gerais, Programa de Pós-graduação em Periodontia (Belo Horizonte/MG, Brazil).; 3 Pontifícia Universidade Católica de Minas Gerais, Faculdade de Odontologia (Belo Horizonte/MG, Brazil).

**Keywords:** Cleft lip and palate, Maxillary expansion, Plaque index, Gingival index

## Abstract

**Objective:**

To assess, during rapid maxillary expansion, the plaque index (PI) and the gingival index (GI) of patients with unilateral cleft lip and palate(UCLP) using Hyrax (HX) or inverted mini-Hyrax (IMHX) rapid maxillary expanders (RME) considering patients’ sex and age.

**Methods:**

PI (Quigley Index modified by Turesky et al) and GI (Löe and Silness) of 28 UCLP (11 females; 17 males: aged 8 to 15 years) submitted to daily RME activation were assessed before (T_0_) and 7 (T_1_), 28 (T_2_) and 90 (T_3_) days after activation. Log-linear models and Bonferroni correction were performed to analyze possible differences in PI and GI between RME, sexes or age groups over time.

**Results:**

Intra-group comparison revealed significant increases in PI of patients using HX (T_0_ < T_2_), IMHX (T_0_ < T_3_; T_1_< T_3_), males (T_0_ < T_1_; T_0_ < T_2_; T_0_ < T_3_) or aged 12-15 years (T_0_ < T_1_; T_0_ < T_2_; T_0_ < T_3_), and in GI of patients using IMHX (T_0_ < T_3_; T_1_ < T_3_), females (T_1_ < T_3_; T_2_ < T_3_) or aged 12-15 years (T_0_ < T_3_; T_2_ < T_3_). One inter-group difference in GI according to patients’ age (8-11 < 12-15; T_1_) was observed.

**Conclusions:**

Since a single difference between groups was encountered, the results of this study indicated that PI and GI during maxillary expansion were similar between HX and IMHX, sexes and the analyzed age groups. Therefore, orthodontists can use these RME in UCLP patients according to the patient’s necessity or their preferences.

## RESUMO

**Objetivo:**

avaliar, durante a expansão rápida da maxila (ERM), o índice de placa dentária (IP) e o índice gengival (IG) de pacientes com fissura labiopalatina unilateral (FLPU), usando o aparelho disjuntor Hyrax (HX) ou o mini-Hyrax invertido (MHXI), considerando-se também o sexo e a idade.

**Métodos:**

o IP (Quigley-Hein modificado por Turesky et al.) e o IG (Silness e Löe) de 28 pacientes com FLPU (11 meninas; 8-15 anos de idade) tratados com ativação diária do aparelho de ERM foram avaliados antes (T_0_) e após 7 (T_1_), 28 (T_2_) e 90 (T_3_) dias da ativação inicial. Modelos de regressão *log*-linear e correção de Bonferroni foram usados para analisar as possíveis diferenças de IP e IG entre os grupos, de acordo com o tipo de aparelho de ERM, sexo e idade, ao longo do tempo.

**Resultados:**

as comparações intragrupos revelaram aumentos significativos do IP em pacientes usando HX (T_0_< T_2_) ou MHXI (T_0_< T_3_; T_1_< T_3_), com idades entre 12 e 15 anos (T_0_ < T_1_; T_0_ < T_2_; T_0_ < T_3_), e do IG de pacientes usando MHXI (T_0_< T_3_; T_1_< T_3_), que eram meninas (T_1_ < T_3_; T_2_ < T_3_), com idades entre 12 e 15 anos (T_0_ < T_3_; T_2_ < T_3_). Uma diferença no IG entre grupos foi observada em relação à idade dos pacientes (8-11 < 12-15; T_1_).

**Conclusões:**

uma única diferença entre grupos foi encontrada, o que sugere que os resultados de IP e IG, durante a expansão maxilar, foram semelhantes entre os grupos HX e MHXI, bem como entre os sexos e idades analisadas. Dessa forma, os ortodontistas podem usar esses aparelhos de ERM no tratamento de pacientes com FLPU de acordo com as necessidades do paciente ou suas preferências.

## INTRODUCTION

Cleft lip and palate (CLP) is the most common congenital malformation of the craniofacial region, affecting the subjects’ midface and limiting proper maxillary growth.^1^ ^,^ ^2^ CLP occurs in approximately 1 in every 700 live births and compromises the quality of life of more than 7.5 million children around the world.^3^ In addition to problems during midface development, some studies^4-7^ have reported that children with CLP present with poor oral hygiene and increased prevalence of caries and periodontal disease, compared with unaffected children. The difficulty in maintaining proper oral hygiene may be due to the anatomy of the cleft area, the presence of scar tissue from previous surgical treatments, decreased interest of CLP patients in achieving adequate oral hygiene and the apprehensions that CLP children often have when brushing the teeth adjacent to the cleft region.^5^ ^,^ ^6^

The scar tissues in areas of the palate of CLP patients not only affect oral hygiene but also compromise the transversal and sagittal growth of the maxilla.^8^ ^,^ ^9^ The resultant decrease in the transverse dimension of the arch, particularly in the anterior region,^10^ indicates that rapid maxillary expansion is necessary to correct the transverse maxillary deficiencies that are frequently observed in the upper arches of CLP patients.^11^ ^,^ ^12^ This expansion can be achieved through different methods,^13^ ^,^ ^14^ including the use of rapid maxillary expanders (RME),^15^ such as the Hyrax (HX) and the inverted mini-Hyrax (IMHX) appliances.^16^

The use of fixed orthodontic appliances, RME and other orthodontic devices increase the challenges for maintaining adequate oral hygiene and thus increase the likelihood that orthodontic patients present higher plaque levels, more caries, gingivitis and periodontal problems.^4^ ^-^ ^6^ ^,^ ^17^ Since biofilm control is particularly important for patients with CLP, and HX and IMHX expanders present different structures, the present study investigated whether using these two different RMEs resulted in significant differences in the plaque index (PI) and gingival index (GI) during maxillary expansion in CLP patients.

## MATERIAL AND METHODS 

### Sample selection 

This study was independently reviewed and approved by the Institutional Review Board of Pontifical Catholic University of Minas Gerais (PUC-MG). Written informed consent was obtained from all of the participants and their parents/guardians. The following inclusion criteria were used: presence of unilateral CLP and maxillary transverse deficiency requiring expansion; absence of syndromes and/or systemic problems that would contraindicate the proposed treatment and no previous orthodontic interventions. The primary objective of this study was to compare the potential differences in PI and GI scores during rapid maxillary expansion therapy with two different RME (i.e., the HX and IMHX). A sample size calculation was performed based on the primary outcome of PI and GI data from a prior study^18^ considering a significance level of 5%, a power of 80%, and 15% minimum differences between groups in the PI and GI scores (mean values). The results indicated that 12 subjects in each group were required for the study and that including 14 in each group would be safe, considering a subject dropout rate of 20%. Therefore, a total of 28 patients (11 females and 17 males; aged between 8-15 years; mean age of 11.3 years) were selected among individuals who sought orthodontic treatment at the Center for Treatment of Craniofacial Anomalies at PUC-MG.

### Orthodontic procedures

**Figure 1:**
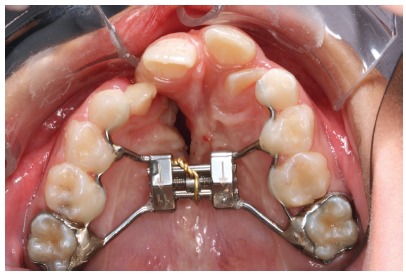
Hyrax expander.

According to the transverse deficiency extension subjects were allocated in specific groups. The individuals with anterior and posterior maxillary constriction (5 females and 9 males) received the HX expander and those with a more severe anterior maxillary constriction (6 females and 8 males), the IMHX expander. The initial clinical procedures included anamnesis, oral prophylaxis and instructions about how to maintain adequate oral hygiene during orthodontic treatment. The HX presented a jackscrew (Leone Orthodontics and Implantology, Firenze, Italy) in the median region of the expander and two segments of stainless steel wire that followed the palatal surface of the crown of the first and second premolars and/or the first and second deciduous molars. Bands were placed on the first permanent molars and screw arms were attached to the two segments by welding (Fig 1). The HX expander arms were bonded to each tooth with composite to increase appliance stability.

**Figure 2:**
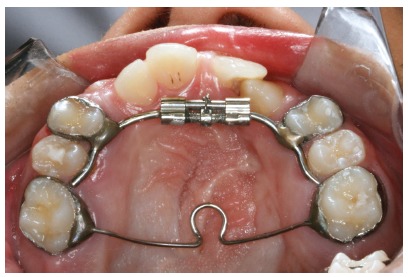
Inverted mini-Hyrax expander.

The IMHX presented a jackscrew (Variety Expander, Dentaurum, Saint Ann, MO, USA) in the anterior region with arms that were bent posteriorly and soldered bilaterally to the first premolar bands. The extensions from the expander screw followed the palatal surface of the crowns of the first and second premolars and/or the first and second deciduous molars. A fixed transpalatal arch was connected the maxillary first molars to prevent posterior expansion, and stainless steel rods were welded on prior to appliance insertion to incorporate the canines into the expansion (Fig 2).

Each expander was cemented with a fluoride-releasing cement (Ultra Band-Lock; Reliance Orthodontic Products, Itasca, IL, USA), and the same laboratory technician fabricated all of the expanders. Daily activation was conducted until the tip of the lingual cusps of the maxillary teeth touched the tips of the buccal cusps of the mandibular teeth. The expanders were used for three months to maintain the obtained transverse correction and immediately after their removal, transpalatal bars with anteriorly extended arms were installed to maintain the transverse improvements.

### Plaque index and gingival index 

The PI and GI of the participants were assessed at different stages. The first measurement was performed without the RME (T_0_), and the subsequent measurements occurred at 7 (T_1_), 28 (T_2_) and 90 (T_3_) days after the RME insertion. The Quigley Index modified by Turesky et al^19^ was used to evaluate the PI. Briefly, the tooth surfaces were stained with 2.0% Erythrosin highlighter, and scores of 0 to 5 were recorded for the buccal and lingual surfaces. In this scoring system, 0 indicated no visible plaque, and 5 indicated that more than 2/3 of the tooth surface was covered in plaque. Teeth that were banded and teeth surfaces that supported orthodontic wires were not evaluated in order to avoid inappropriate analysis.^20^ The Löe and Silness gingival index^21^ was adopted. This index involves a scale from 0 to 3 for the buccal, lingual, mesial and distal surfaces that is scored as follows: 0 indicates healthy gums; 1 indicates slight color changes, light edema and no presence of bleeding on probing; 2 indicates edema with slight redness and bleeding on probing; and 3 indicates severe edema, redness, the presence of ulceration and a tendency for spontaneous bleeding.

### Statistical analysis

To analyze whether differences in the two RME’s, sex or age significantly influenced the PI and GI over time, log-linear marginal models were adjusted, and the backward method for variable selection was applied based on deviation analyses with the χ^2^ test. For the final models that exhibited significant interactions, the Holm-Bonferroni method was used for multiple comparisons. The marginal models, also known as Generalized Equations Estimating (GEE) method, can be considered as an extension of the Generalized Linear Models that allow investigating possible correlations between measurements taken in the same individual/tooth. Due to its simple interpretation and lack of distributional assumptions it is preferred as an extension of the Generalized Linear Models for longitudinal data. A 5% level of significance was adopted, and R software version 3.0.1 was used (www.R-project.org).

## RESULTS

The PI and GI results stratified by RME are described in Table 1. Increase in the mean PI values occurred from T_0_ to T_3_ with both HX and IMHX devices. Intra-group comparisons revealed specific significant increases in the patients who used the HX (T_2_> T_0_; *p*= 0.041) and the IMHX (T_3_> T_0_: *p*= 0.023; T_3_> T_1_: *p*< 0.001). Increases in the mean GI values also occurred from T_0_ to T_3_ in the HX and IMHX groups. Intra-group comparisons revealed specific significant increases in the patients who used the IMHX (T_3_> T_0_: *p*= 0.025; T_3_> T_1_: *p*= 0.003). Inter-group comparisons did not detect significant differences in the PI or GI at any specific time between the HX and IMHX groups.

**Table 1:** Plaque index and gingival index stratified by rapid maxillary expander (RME).

	Variables		T_0_		T_1_		T_2_		T_3_	
			Mean	SE	Mean	SE	Mean	SE	Mean	SE
Plaque Index	RME	HX	2.283^b^	0.050	2.559	0.057	2.723^b^	0.054	2.528	0.053
		IMHX	2.210^c^	0.048	2.493^d^	0.057	2.685	0.058	2.796^c.d^	0.055
Gingival Index	RME	HX	1.220	0.021	1.340	0.019	1.320	0.016	1.370	0.017
		IMHX	1.040^c^	0.023	1.250^d^	0.018	1.300	0.019	1.400^c.d^	0.018

T_0_ = baseline; T_1_ = 7 days; T_2_ = 28 days; T_3_ = 90 days; SE = standard error; RME = rapid maxillary expander; HX = Hyrax; IMHX = inverted mini-hyrax. Significant (*p*< 0.05) intra-group comparisons: a (T_0_ x T_1_); b (T_0_ x T_2_); c (T_0_ x T_3_); d (T_1_ x T_3_).

Since previous studies have reported that sex^22^ and increases in age^6^ among young patients can influence PI and GI results, these factors were also analyzed in the present study. Increases in the mean PI values occurred from T_0_ to T_3_ in the 11 females and 17 males, and in the patients aged 8-11 (6 females and 11 males) and 12-15 years (5 females and 6 males), as described in Table 2. Intra-group comparisons revealed specific significant increases in the males (T_1_ >T_0_: *p*=0.007; T_2_ >T_0_: *p*<0.001; T_3_ >T_0_: *p*<0.001) and in the patients aged 12-15 years (T_1_ >T_0_: *p*=0.009; T_2_ >T_0_:*p*<0.001; T_3_ >T_0_: *p*<0.001). Again, an increase in the mean GI values occurred from T_0_ to T_3_. Intra-group comparisons revealed specific significant increases among the females (T_3_ >T_1_: *p*=0.021; T_3_ >T_2_: *p*=0.046) and among the patients aged 12-15 years (T_3_ >T_0_:*p*=0.011; T_3_ >T_2_: *p*=0.044). Inter-group comparisons did not detect significant PI differences at any specific time between the males and females, as well as between the patients aged 8-11 and 12-15 years. In contrast, inter-group comparisons detected a unique significant difference in the GI results according to age (12-15 > 8-11: *p*= 0.002) at T_1_.

**Table 2:** Plaque index and gingival index stratified by sex and age.

	Variables		T_0_		T_1_		T_2_		T_3_	
			Mean	SE	Mean	SE	Mean	SE	Mean	SE
Plaque Index	Sex	F	2.281	0.058	2.246	0.056	2.441	0.061	2.353	0.058
		M	2.224^a.b.c^	0.043	2.710^a^	0.054	2.877^b^	0.051	2.869^c^	0.049
	Age	8-11	2.370	0.046	2.460	0.052	2.620	0.056	2.600	0.052
		12-15	2.080^a.b.c^	0.051	2.620^a^	0.062	2.810^b^	0.055	2.750^c^	0.056
Gingival Index	Sex	F	1.180	0.024	1.340^d^	0.020	1.380^e^	0.02	1.430^d.e^	0.021
		M	1.110	0.021	1.270	0.017	1.270	0.016	1.350	0.015
	Age	8-11	1.160	0.022	1.210^f^	0.019	1.270	0.018	1.310	0.017
		12-15	1.110^c^	0.023	1.380^f^	0.018	1.350^e^	0.017	1.450^c.e^	0.018

T_0_ = baseline; T_1_ = 7 days; T_2_ = 28 days; T_3_ = 90 days; SE = standard error; F = female; M = male; Significant (*p*< 0.05) intra-group comparisons: a (T_0_ x T_1_); b (T_0_ x T_2_); c (T_0_ x T_3_); d (T_1_ x T_3_); e (T_2_ x T_3_); Significant (*p* < 0.05) inter-group comparison: f (8-11 x 12-15).

## DISCUSSION

Previous studies^4^ ^-^ ^6^ have reported that patients with CLP present increased levels of gingivitis and poorer oral hygiene during orthodontic treatment than do matched controls. Other studies^22^ ^,^ ^23^ that have assessed biofilm and periodontal alterations in non-cleft orthodontic patients have also reported significant increases in these parameters. Oral hygiene during rapid or slow maxillary expansion^18^ and the effects of the use of electric versus manual toothbrushes^24^ on plaque accumulation during rapid maxillary expansion have also been previously evaluated. Furthermore, differences in oral hygiene between young male and female patients^22^ and tendencies toward increases in PI and GI with age (i.e., children versus adolescents) in CLP patients^6^ have been described. However, to date, there are no studies in the literature that have compared the effects of different types of RME on PI and GI or the influences of the sex or age of CLP patients on these parameters during rapid maxillary expansion. Therefore, the present study was conducted. 

The absence of PI and GI significant differences at T_0_(baseline; Table 1) for subjects in HX and IMHX groups demonstrate that HX and IMHX groups were initially homogeneous in this regard. No significant differences in the PI of the buccal and lingual tooth surfaces were observed in the present study. A previous study that evaluated patients without CLP who used acrylic appliances reported that the intraoral locations of biomaterials influence the formation of *in situ* biofilms because the use of appliances promotes a reduction in the biofilm thicknesses on the palatal tooth surfaces.^25^ According to these authors, such reductions might be due to increased tongue activity, which would result in a mechanism of self-cleaning the palatal surfaces. Another study performed with non-cleft patients reported that the use of fixed customized lingual appliances promoted increases in biofilm accumulation and gingival inflammation in the lingual region.^23^ The opposing results of these two studies may be due to the use of different methods for biofilm evaluation and the use of different orthodontic appliances.

Although the PI increased over time in the patients who used the HX (T_0_: 2.283 ± 0.050; T_3_: 2.528 ± 0.053) and the IMHX (T_0_: 2.210 ± 0.048; T_3_: 2.796 ± 0.053), significant differences between the PI of these RME were not registered, and differences were observed only in specific intra-group comparisons. The PI analyses by sex and age revealed similar patterns because the females (T_0_: 2.281 ± 0.058; T_3_: 2.353 ± 0.058), males (T_0_: 2.224 ± 0.043; T_3_: 2.869 ± 0.049), patients aged 8-11 (T_0_: 2.370 ± 0.046; T_3_: 2.600 ± 0.052) and 12-15 years (T_0_: 2.080 ± 0.051; T_3_: 2.750 ± 0.056) exhibited an increase in PI over time, and significant differences in specific intra-group comparisons. When the same analyses were conducted to evaluate the GI, again a similar pattern was observed since the GI increased over time in the patients who used HX (T_0_: 1.220 ± 0.021; T_3_: 1.370 ± 0.017), IMHX (T_0_: 1.040 ± 0.023; T_3_: 1.400 ± 0.018), in females (T_0_: 1.180 ± 0.024; T_3_: 1.430 ± 0.021), males (T_0_: 1.110 ± 0.021; T_3_: 1.350 ± 0.015), patients aged 8-11 (T_0_: 1.160 ± 0.022; T_3_: 1.310 ± 0.017) and 12-15 years (T_0_: 1.110 ± 0.023; T_3_: 1.450 ± 0.018). Therefore, significant differences were observed in specific intra-group comparisons, but in contrast to the PI results, a unique significant (*p*= 0.002) inter-group difference(8-11 < 12-15) at T_1_ was encountered.

These results are similar to those of previous studies that have reported plaque increases during orthodontic treatment in patients with^4^ ^-^ ^6^ or without^23^ CLP during orthodontic treatment. However, in contrast to one previous study,^22^ the present study did not detect effects of sex on the PI or GI, a difference that might have resulted from the absence of orthodontic devices and CLP patients in the previous study. From T_1_ to T_3_, the patients aged from 12 to 15 years exhibited higher PI and GI values than the patients aged 8-11 years. These results are similar to another study that also associated increases in PI and GI with age rise in young CLP patients^6^ undergoing orthodontic treatments.

Finally, to the best of our knowledge, this is the first study to conduct these specific analyses. The PI and GI exhibited significant increases in specific groups over time, and because a unique significant difference between groups was encountered, the results of the present study indicate that the influences of these RMEs on the PI and GI of male and female UCLP patients aged 8-15 years are similar during rapid maxillary expansion.

## CONCLUSION

Although the plaque index and gingival index of patients with unilateral cleft lip and palate increased during rapid maxillary expansion, important differences between rapid maxillary expanders, sexes or age during this procedure were not observed. Therefore, orthodontists can use these RME in UCLP patients according to the patient’s clinical needs or their own clinical preferences.
